# Prediction of Dengue Disease Severity among Pediatric Thai Patients Using Early Clinical Laboratory Indicators

**DOI:** 10.1371/journal.pntd.0000769

**Published:** 2010-08-03

**Authors:** James A. Potts, Robert V. Gibbons, Alan L. Rothman, Anon Srikiatkhachorn, Stephen J. Thomas, Pra-on Supradish, Stephenie C. Lemon, Daniel H. Libraty, Sharone Green, Siripen Kalayanarooj

**Affiliations:** 1 Center for Infectious Disease and Vaccine Research, University of Massachusetts Medical School, Worcester, Massachusetts, United States of America; 2 Department of Virology, Armed Forces Research Institute of Medical Sciences, Bangkok, Thailand; 3 Queen Sirikit National Institute of Child Health, Bangkok, Thailand; 4 Division of Preventive and Behavioral Medicine, University of Massachusetts Medical School, Worcester, Massachusetts, United States of America; Pediatric Dengue Vaccine Initiative, United States of America

## Abstract

**Background:**

Dengue virus is endemic in tropical and sub-tropical resource-poor countries. Dengue illness can range from a nonspecific febrile illness to a severe disease, Dengue Shock Syndrome (DSS), in which patients develop circulatory failure. Earlier diagnosis of severe dengue illnesses would have a substantial impact on the allocation of health resources in endemic countries.

**Methods and Findings:**

We compared clinical laboratory findings collected within 72 hours of fever onset from a prospective cohort children presenting to one of two hospitals (one urban and one rural) in Thailand. Classification and regression tree analysis was used to develop diagnostic algorithms using different categories of dengue disease severity to distinguish between patients at elevated risk of developing a severe dengue illness and those at low risk. A diagnostic algorithm using WBC count, percent monocytes, platelet count, and hematocrit achieved 97% sensitivity to identify patients who went on to develop DSS while correctly excluding 48% of non-severe cases. Addition of an indicator of severe plasma leakage to the WHO definition led to 99% sensitivity using WBC count, percent neutrophils, AST, platelet count, and age.

**Conclusions:**

This study identified two easily applicable diagnostic algorithms using early clinical indicators obtained within the first 72 hours of illness onset. The algorithms have high sensitivity to distinguish patients at elevated risk of developing severe dengue illness from patients at low risk, which included patients with mild dengue and other non-dengue febrile illnesses. Although these algorithms need to be validated in other populations, this study highlights the potential usefulness of specific clinical indicators early in illness.

## Author Summary

Patients with severe dengue illness typically develop complications in the later stages of illness, making early clinical management of all patients with suspected dengue infection difficult. An early prediction tool to identify which patients will have a severe dengue illness will improve the utilization of limited hospital resources in dengue endemic regions. We performed classification and regression tree (CART) analysis to establish predictive algorithms of severe dengue illness. Using a Thai hospital pediatric cohort of patients presenting within the first 72 hours of a suspected dengue illness, we developed diagnostic decision algorithms using simple clinical laboratory data obtained on the day of presentation. These algorithms correctly classified near 100% of patients who developed a severe dengue illness while excluding upwards of 50% of patients with mild dengue or other febrile illnesses. Our algorithms utilized white blood cell counts, percent white blood cell differentials, platelet counts, elevated aspartate aminotransferase, hematocrit, and age. If these algorithms can be validated in other regions and age groups, they will help in the clinical management of patients with suspected dengue illness who present within the first three days of fever onset.

## Introduction

Dengue fever (DF) and dengue hemorrhagic fever (DHF), the more severe form of dengue illness, are re-emerging viral diseases [1]. Dengue is endemic in countries in tropical and subtropical areas. Dengue viruses are transmitted through the bite of an infected mosquito [2]. Illnesses caused by dengue viruses can range from a nonspecific febrile illness, as in most DF cases, to more severe illness with bleeding, thrombocytopenia, and plasma leakage, in cases of DHF [3]. DHF with circulatory failure defines DHF grades 3 and 4, also termed dengue shock syndrome (DSS) [3]. However, strict adherence to WHO criteria for diagnosis of DHF has been difficult and some researchers have established different categories of severe dengue illnesses [4]–[7].

Dengue has a substantial economic impact in developing countries [8], [9]. Individuals and families are impacted by lost wages, cost of seeking care, cost of treatment, missed school, and extended effects of recovery [8]–[12]. Prevention and control strategies have been poorly implemented or unsustained and thus largely ineffective [13], [14].

Currently, there is no licensed vaccine or anti-viral against dengue. The treatment for patients with suspected dengue is supportive care consisting of rehydration and anti-pyretics [3]. Patients with suspected dengue are often hospitalized for close monitoring. Plasma leakage occurs around the time of defervescence. Prior to this critical phase, it has proven difficult to differentiate mild vs. severe dengue illness. Ideally, only severe cases of DF and DHF should be hospitalized. However, there are no diagnostic/prognostic tools available to distinguish severe dengue from non-severe dengue or other febrile illness (OFI) at early stages of illness. Such tools could improve clinical practice by decreasing the number of un-needed hospitalizations, improving utilization of limited hospital resources to treat more severely ill patients, improving outcomes of severely ill patients by administering needed care earlier, and improving the capability of physicians in developing or rural areas to make a more accurate early diagnosis.

We conducted a prospective study of Thai children with acute febrile illness, consistent with dengue, enrolled from an early stage of illness onset [15]. We applied classification and regression tree (CART) analysis to this dataset to distinguish patients with severe dengue illness from those with mild dengue illness and OFI. CART was used to establish a diagnostic decision tree using clinical laboratory variables and patient characteristics collected at presentation.

## Methods

### Study Setting

A longitudinal observational study was conducted at two hospitals in Thailand: (1) the Queen Sirikit National Institute of Child Health (QSNICH) in Bangkok during 1994–97, 1999–2002, and 2004–07, and (2) the Kamphaeng Phet Provincial Hospital (KPPH) in the Kamphaeng Phet providence in a rural northern section of Thailand during 1994–97. The study methods have been described in detail elsewhere [15]. In brief, children between the ages of six months and 15 years presenting with temperature ≥38°C for no more than 72 hours and no localizing symptoms were identified in the outpatient department or on the hospital ward were eligible for enrollment with parental consent. Exclusion criteria included: signs of shock at presentation, chronic illness, or an initial alternate non-dengue diagnosis. All subjects were admitted to the hospital and monitored until 24 hours after defervescence. Fluid management (oral rehydration or intravenous fluid) was guided by the ward physician based upon clinical necessity. The study protocol was approved by the Institutional Review Boards of all participating institutions.

A blood sample was obtained on the day of enrollment and daily thereafter until discharge or for a maximum of five consecutive blood collections. Serological assays (IgM/IgG ELISA and hemagglutination inhibition assay), virus isolation, and/or RT-PCR were used to confirm all dengue cases. Patients were observed and daily clinical and laboratory measurements were recorded using standardized data collection forms.

After defervescence (2 consecutive temperatures below 38°C), serial finger-stick hematocrits were measured to capture hemoconcentration. A right lateral decubitus chest x-ray was taken the day following defervescence and a pleural effusion index (PEI) was measured as 100×(maximum width of right pleural effusion)/(maximum width of right hemithorax). After completion of the case record, a single expert physician (author S.N.), who was not directly involved in patient care, assigned a final diagnosis of DF, DHF, or OFI based upon chart review following WHO guidelines [3].

### Categories of Dengue Illness Severity

Given that not all DHF cases are severe and not all DF cases are mild, we applied several different categories of dengue disease severity using data from each patient's entire hospital course : (1) dengue shock syndrome (DSS, as defined by WHO criteria); (2) DSS or PEI>15; (3) DSS or required intravenous fluid; (4) DSS or platelet count < = 50,000 anytime during illness; (5) DSS or received fluid intervention (oral or intravenous) in any 24-hour period that exceeded maintenance volume +5% volume deficit [16], [17].

### Clinical Laboratory Variables and Patient Characteristics

The input variables used for establishing each tree were platelet count, hematocrit, WBC count, percent monocytes, percent lymphocytes, percent neutrophils, AST, ALT, tourniquet test (+/−), age, and gender, all of which were obtained on the day of presentation.

### Statistical Analysis

Descriptive characteristics of the study sample were compared using t-tests and Pearson's χ^2^. CART analysis was performed using SPSS Answer Tree 3.0 software (see Text S1) [18]. CART analysis is a non-parametric analytic tool that has many advantages over logistic regression models, such as its ability to detect interaction between variables used to generate a tree [19], [20]. Age, gender, and clinical laboratory data on the day of presentation were used to establish diagnostic decision trees to distinguish between patients with severe dengue illness and those with non-severe illness or OFI. Stopping rules were: (1) no terminal node could contain <5% of the original sample size, (2) no more than 5 levels per tree, and (3) a minimum improvement in impurity of .0001.

Additional analyses were performed to examine differences in diagnostic trees according to the day of presentation among the low risk, non-severe group. The final trees selected were those that had minimum misclassification of severe dengue illness in low risk nodes (high sensitivity) and maximum correct classification of non-severe dengue and OFI in low risk nodes (high specificity). In each terminal node, patients were classified as low risk or elevated risk of severe dengue illness where optimal sensitivity could be achieved. For all analyses, sensitivity was weighted more heavily than specificity by using misclassification cost ratio of 1∶10 severe dengue vs. non-severe. Each tree was validated using the k-fold cross validation method [21], [22]. We used k = 5 in our analysis.

## Results

### Study Sample

In total, 1384 patients were enrolled in the study. Of these, 1311 had a final diagnosis of DHF, DF, or OFI. Of the remaining 73 patients, 32 had an undetermined diagnosis due to lack of convalescent blood sample for serology, and 41 had a presumed non-viral infection. An additional 81 patients (28 DHF, 20 DF, and 33 OFI) were missing one or more variables of interest on the day of presentation and were excluded from the analysis. Table 1 describes the 1230 patients included in the analysis. Among these, 208 had a final physician diagnosis of DHF (53 grade 1, 118 grade 2, 36 grade 3, 1 grade 4), 374 had DF, and 648 had OFI. Secondary infections accounted for 81.9% of all dengue infections (74.6% of DF cases and 95.2% of DHF cases). The majority of dengue infections were DENV1 (40.7%). Table 2 indicates the number of patients with severe dengue based on different definitions as well as how trees produced with each of the definitions (Outcome variable for tree) performed when severity was defined differently (Outcome variable for evaluation of tree).

**Table 1:** Study sample characteristics, in the total sample and by final diagnosis.

	Age (mean, 95% CI, years)	Gender (m∶f ratio)	Days ill at presentation mean (median)	Length of observational period (24 hours after defervescence) mean(median)*
DHF				
Grade 1 (n = 53)	8.4 (7.4, 9.4)	2.3	2.0 (2)	6.2 (6)
Grade 2 (n = 118)	9.1 (8.5, 9.6)	1.3	2.3 (2)	6.4 (6)
Grade 3/4	8.5 (7.6, 9.4)	0.9	2.4 (2)	7.3 (7)***
(n = 37)**				
DF (n = 374)	8.6 (8.4, 8.9)	1.1	2.1 (2)	6.3 (6)
OFI (n = 648)	7.1 (6.8, 7.3)	1.2	1.8 (2)	5.3 (5)

*Includes only those patients who remained in the study until the end of the observational period (50 DHF grade1; 110 DHF grade 2; all DHF grade 3 and 4; 327 DF; 495 OFI).

**Only 1 patient had DHF grade 4; this subject was combined with DHF grade 3 for analysis.

***DHF grade 3 or 4 had longer observational periods when compared to patients with DHF grade 1 and 2, DF, or OFI (p<.001).

**Table 2:** CART analysis using different categories of severe dengue illness.*

Tree	Outcome variable for generating tree**	Outcome variable for evaluation of tree†	% Misclassified severe dengue (# classified as low risk/total severe)¶	% Correctly classified non- severe (# classified as low risk/total non-severe)¶¶
1	Severity Category 1	Severity Category 1	2.7%	48.3%
			(1/37)	(576/1193)
		Severity Category 2	16.9%	49.1%
			(14/83)	(563/1147)
		Severity Category 3	16.8%	51.1%
			(25/149)	(552/1081)
		Severity Category 4	12.5%	52.1%
			(20/160)	(557/1070)
		Severity Category 5	17.6%	48.6%
			(12/68)	(565/1162)
2	Severity Category 2	Severity Category 1	0.0%	42.2%
			(0/37)	(504/1193)
		Severity Category 2	1.2%	44.0%
			(1/83)	(505/1147)
		Severity Category 3	9.4%	45.5%
			(14/149)	(492/1081)
		Severity Category 4	5.0%	46.5%
			(8/160)	(498/1070)
		Severity Category 5	8.8%	43.0%
			(6/68)	(500/1162)
3	Severity Category 3	Severity Category 3	34.9%	72.1%
			(52/149)	(779/1081)
4	Severity Category 4	Severity Category 4	42.5%	81.5%
			(68/160)	(872/1070)
5	Severity Category 5	Severity Category 5	42.6%	77.3.0%
			(29/68)	(898/1162)

*Severity Category 1: DSS (DHF grade 3 or 4).

Severity Category 2: DSS or PEI>15.

Severity Category 3: DSS or required intravenous fluid resuscitation during hospitalization.

Severity Category 4: DSS or had min platelet count < = 50,000 during hospitalization.

Severity Category 5: DSS or received fluid intervention (oral or intravenous) >5% volume deficit above maintenance.

**Severity category used to generate tree.

**†:** Severity category used to evaluate the tree that was generated using another severity category (Column 2).

**¶:** “Severe dengue” refers to criteria in Column 3.

**¶¶:** “Non-severe dengue” refers to criteria in Column 3.

### Classification Tree for Dengue Shock Syndrome

**Figure 1:**
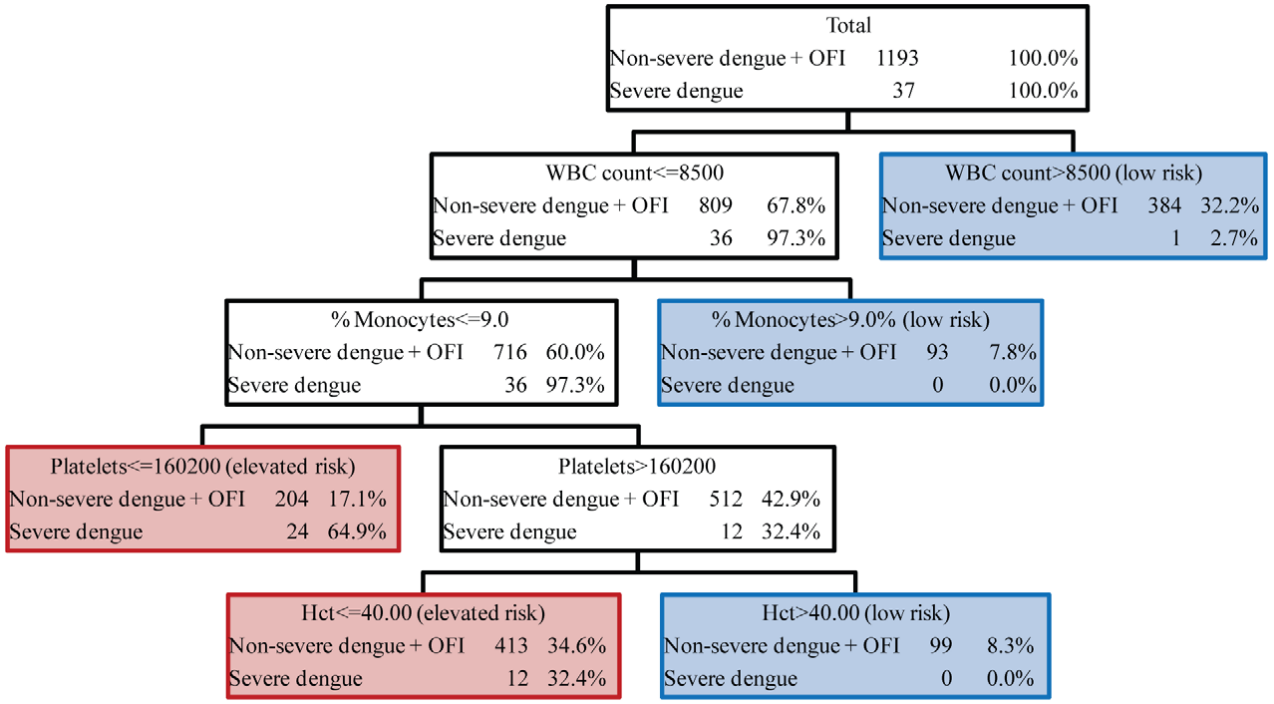
CART algorithm #1 for identifying patients who subsequently developed severe dengue (defined as WHO criteria for dengue shock syndrome, DSS) using clinical laboratory data obtained within the first three days of illness. Each node is shown with the selected splitting variable, the number of patients with severe/non-severe or OFI, and the proportion of each from the parent node. Terminal nodes are marked as ‘elevated risk’ of severe dengue illness, outlined in red, and ‘low risk’ of severe dengue, outlined in blue.

Trees were generated for each of the five categories of severe dengue illness. As summarized in Table 2 and shown in Figure 1 (Tree 1), the tree that provided the best distinction on the day of presentation categorized severe dengue as DSS. The initial splitting variable in the tree is WBC count; other variables in the tree include percent monocytes, platelet count, and hematocrit. The tree resulted in five terminal nodes, of which three are considered low risk and two are considered elevated risk. The three low risk nodes are 1) WBC>8500, 2) WBC< = 8500 and percent monocytes >9.0, and 3) WBC< = 8500, percent monocytes< = 9.0, platelet count >160200, and hematocrit>40%. The two nodes considered elevated risk of severe dengue were 1) WBC< = 8500, percent monocytes < = 9.0, and platelet count < = 160200 (64.9% of patients with severe dengue) and 2) WBC< = 8500, percent monocytes< = 9.0, platelet count >160200, and Hct< = 40 (32.4% of patients with severe dengue).

A total of 576 (48.3%) patients with non-severe dengue are classified correctly in the low risk group at the cost of misclassifying one patient who later manifested DHF grade 3. The initial splitting variable correctly classified 384 (32%) of the patients with non-severe dengue. The patients that were correctly classified as low risk included 63.7% of all OFI, 32.1% of all DF, 41.5% of all DHF grade 1, and 17.8% of all DHF grade 2. Patients with non-severe dengue illness were more likely than patients with OFI to be classified as elevated risk of severe dengue (70.1% of non-severe dengue versus 36.3% of OFI).

Among the 617 (51.7%) patients with non-severe illness that were classified as elevated risk, the median day of presentation was 72 hours after illness onset and the average length of hospital stay was 6.8 days; patients with non-severe dengue that were correctly classified had a median day of presentation of 48 hours after illness onset and an average length of hospital stay of 7.3 days. To assess differences according to the day of presentation, the tree was applied using data from patients with non-severe illness at 72 hours among patients who were still febrile. In this group of low risk patients, the percent correctly classified as low risk decreased slightly from 48% to 44% (data not shown).

### Classification Tree Using DSS or PEI>15

**Figure 2:**
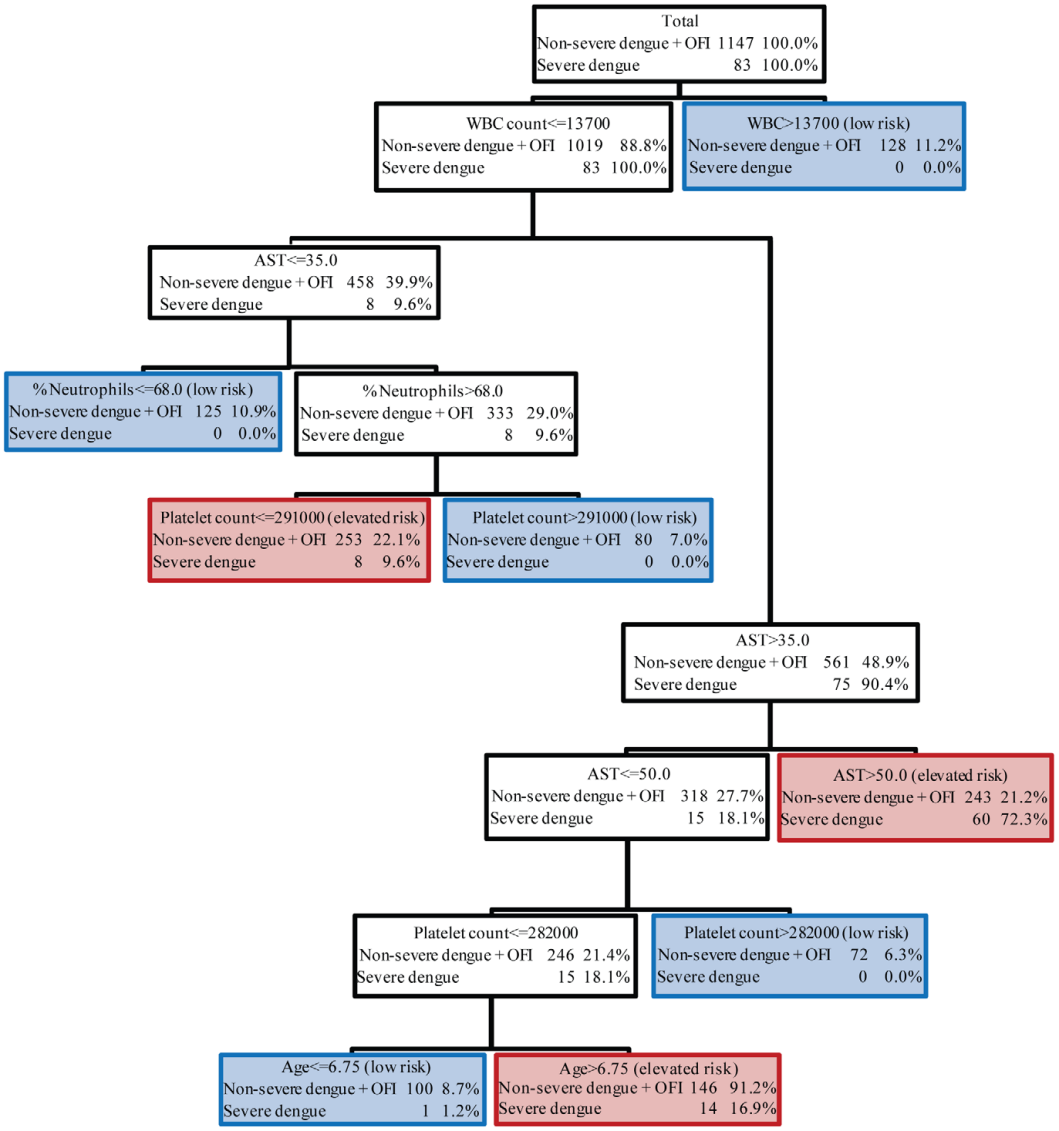
CART algorithm #2 for identifying patients who subsequently developed severe dengue (defined as WHO criteria for dengue shock syndrome, DSS, or dengue with significant pleural effusion) using clinical laboratory data obtained within the first three days of illness. Pleural effusion index (PEI)>15 was used as the criterion for significant pleural effusion. Each node is shown with the selected splitting variable, the number of patients with severe/non-severe or OFI, and the proportion of each from the parent node. Terminal nodes are marked as ‘elevated risk’ of severe dengue illness, outlined in red, and ‘low risk’ of severe dengue, outlined in blue.

Figure 2 shows a diagnostic decision tree in which severe disease was defined as DHF grade 3 or 4 or PEI>15 (Tree 2). This disease categorization added nine patients with DHF grade 1 and 37 patients with DHF grade 2. No patients diagnosed with OFI or DF had a PEI>15. For this tree, the initial splitting variable was WBC count; other variables in the tree include AST, percent neutrophils, platelet count, and age. There are eight terminal nodes, of which five are considered low risk and three are considered elevated risk. The five low risk nodes are 1) WBC>13700, 2) WBC< = 13700, AST 36–50, and platelet count >282000, 3) WBC< = 13700, AST 36–50, platelet count < = 282000, and age< = 6.75, 4) WBC< = 13700, AST< = 35, and percent neutrophils< = 68%, and 5) WBC< = 13700, AST< = 35, percent neutrophils>68%, and platelet count>291000. The three elevated risk nodes are 1) WBC< = 13700, AST>50 (72.3% of patients with severe dengue), 2) WBC< = 13700, AST 36–50, platelet count < = 282000, and age>6.75 (16.9% of patients with severe dengue), and 3) WBC< = 13700, AST< = 35, percent neutrophils>68%, and platelet count< = 291000 (9.6% of patients with severe dengue).

This tree correctly classified 505 (44%) patients with non-severe dengue at the cost of misclassifying one patient with severe dengue. The misclassified patient was diagnosed with DHF grade 2 and had PEI of 25.8. All patients with DHF grade 3 or grade 4 were correctly classified in this tree as elevated risk of severe dengue. Among the 505 patients correctly classified as low risk of severe dengue, 380 were OFI (58.6% of OFI), 105 were DF (28.1% of DF), and 20 were DHF grade 1 or 2 (16.0% of non-severe DHF). Patients with non-severe dengue illness were more likely than patients with OFI to be classified as elevated risk. When the tree was applied using data from patients with non-severe illness at 72 hours, the percent of non-severe cases correctly classified as low risk increased from 44% to 50%.

### Classification Tree Using Other Categories of Dengue Disease Severity

We assessed the generalizability of our trees using other categories of dengue disease severity (Table 2). For example, when applying the tree that was generated using DSS as the only criterion for dengue disease severity (Tree 1) to different categories of severity, the percentage of patients with a severe dengue illness that were misclassified as low risk ranged from 12.5% to 17.6% and the percentage of patients with non-severe illness that were correctly classified ranged from 48.6% to 52.1%. All additional trees (Trees 3–5) had moderate specificity but limited sensitivity (Table 2), with a misclassification of severe dengue as low risk ranging from 34.9% to 42.6% and a correct classification of non-severe illness ranging from 72.1% to 81.5%. Each tree shared the same initial splitting variable of WBC count (data not shown).

## Discussion

Early diagnosis of severe dengue illness not only has the potential to reduce morbidity and mortality, but could also reduce the economic impact of dengue illness by decreasing the duration of hospitalization and the number of patients who will develop shock. We identified two diagnostic algorithms using early clinical laboratory indicators and patient characteristics that could distinguish patients with severe dengue from those with non-severe dengue or other febrile illnesses within the first 72 hours of illness.

When applying these trees to other (broader) categories of disease severity, a high sensitivity was still achieved. Previous studies have shown that modified definitions of dengue disease severity have better agreement with a treating physician's assessment when compared to strict adherence to WHO criteria [4], [7], [23], [24]. For any classification of dengue disease severity utilized, a high proportion of patients with non-severe dengue or other febrile illness were correctly classified as low risk of severe dengue (Table 2). These data suggest that patients classified as ‘elevated risk’ of severe dengue based on these algorithms should be treated and managed more aggressively; in comparison, our data suggest that patients classified as ‘low risk’ of severe dengue could be safely managed on an outpatient basis.

The single patient with severe dengue that was misclassified in Tree 1 presented within the first 24 hours of illness, had an initial WBC count of 13700, and was diagnosed with DHF grade 3. Five other patients with severe dengue in Tree 1 also presented within the first 24 hours and yet were correctly classified as elevated risk. When we further investigated the effect of day of presentation by using day 3 data from all non-severe cases, we found that day of presentation had little effect on the sensitivity of Trees 1 and 2 (within the first 72 hours); Tree 1 still correctly classified 44% of the non-severe cases as low risk of severe dengue infection and, in Tree 2, the percent correctly classified as low risk increased from 44% to 50%.

Many of the variables used in our decision algorithms have been shown to distinguish between patients with dengue and patients with OFI in other settings [25]. Trees 1 and 2 have an initial splitting variable of WBC count, which reinforces the reported utility of this variable in distinguishing severe dengue illness within the first days of illness [25]–[29]. Both trees included nodes using platelet count as the splitting variable. Thrombocytopenia is a hallmark of severe dengue disease, although it frequently occurs in DF as well [3]. Platelet counts are able to distinguish between patients with dengue and OFI [25], [28]. However, when producing a tree using a minimum platelet count of < = 50,000 as part of the categorization of severity (Severity category 4), the tree misclassified 42.5% of patients with severe dengue (Table 2). These data suggest that thrombocytopenia is not a specific marker for severe disease in the early febrile phase of dengue illness.

One criticism of CART analysis is that the cutoff values may not be clinically meaningful. However, when we re-defined the cutoff values for Trees 1 and 2 the results maintained a high sensitivity. For example, in Tree 1 when we rounded platelet count to 160,000, the results remained the same. In Tree 2, when we rounded the cutoffs of platelet count to 290,000 and 280,000, percent neutrophils to 70%, and age to 7, the tree correctly classified 45.9% of the non-severe cases while still achieving 94.0% sensitivity for severe cases.

Interestingly, many of the cutoff lab values in our decision trees fall within the ‘normal’ range; this suggests that established ‘normal’ ranges for routine laboratory tests have low sensitivity to detect clinically relevant changes. For example, some variables, such as hematocrit are normal early in the course of disease but appear to be able to predict those children who will later develop severe disease, often associated with hemoconcentration, as seen in the final node of Tree 1. We interpret this to indicate an interaction with the outcome of severe dengue and the other clinical values used in this Tree.

Tanner and colleagues published an analysis establishing dengue decision trees; however, their analysis was based on only three WHO-defined DHF cases and it was unclear if these three cases met other objective criteria for severity [30]. In contrast, our study has 37 cases of more severe WHO-defined DSS and 171 cases of DHF grade 1 or 2. We also applied other criteria that could classify patients with dengue as having severe illness. Their study included a platelet count of <50,000 as part of the definition of severe dengue, and the resulting tree was limited in its sensitivity (82.6%) [30]. Although the tree had a high specificity, sensitivity is a more important clinical consideration in the detection of severe disease. A more recent decision tree study by Lee and colleagues found a history of clinical bleeding, serum urea and serum protein to distinguish between patients with DF and patients with DHF; however, both studies have limited clinical utility as a predictive algorithm for patients with severe dengue because virologic confirmation of dengue infection is not known at presentation [30], [31]. Our study identifies those with severe dengue illness among all suspected dengue cases.

Our study is subject to some limitations. First, our study included only pediatric patients at two hospitals in Thailand. However, because the majority of dengue cases in Thailand and other regions of Southeast Asia are children, our findings are clinically relevant [3], [32], [33]. Additionally, some patients may have received early fluid intervention as part of standard care which may have modified disease progression and development of severe plasma leakage. Furthermore, because our study enrolled patients only during the initial 72 hours of illness, these algorithms may not adequately reflect clinical practice outside of a research setting where many patients present for medical attention after the first 72 hours of illness. Therefore, we cannot make any conclusions regarding the sensitivity and specificity of these classification trees at later time points in illness. We recognize that clinical algorithms cannot replaced by good clinical management. Further validation using datasets from additional prospective cohort studies conducted in other dengue endemic regions is needed to establish the clinical utility of our algorithms in other populations.

We provide two decision tree algorithms using 12 years of systematically collected clinical data from a well-defined cohort of pediatric patients in a dengue-endemic region. Our algorithms have minimal misclassification of WHO-defined DSS cases among all patients with suspected dengue infection who present within the first 72 hours of illness. These algorithms also have minimal misclassification of other severe dengue illnesses using different categorizations of severity. A robust, validated decision algorithm can be easily implemented in resource limited settings to identify patients who are at risk for developing a more severe dengue illness and limit the number of unneeded hospitalizations.

## Supporting Information

Checklist S1 STROBE checklist
(0.08 MB DOC)

Text S1 Supplementary Methods
(0.02 MB DOC)
