# Supplementary material for: Prediction of Dengue Disease Severity among Pediatric Thai Patients Using Early Clinical Laboratory Indicators
Source: PLoS Negl Trop Dis. 2010 Aug 3;4(8):e769. doi: 10.1371/journal.pntd.0000769 (PMC2914746; doi:10.1371/journal.pntd.0000769)
Supplement: Text S1 — Supplementary Methods (0.02 MB DOC) [file pntd.0000769.s002.doc]

Supplementary Methods

Classification and Regression Tree Analysis

CART is a non-parametric statistical technique that is used to partition data into mutually exclusive and exhaustive subgroups (nodes) where patients within each partitioned node are as homogenous as possible in terms of the outcome measure of interest (diagnosis) [21]. The data is partitioned according to the independent variable that provides the greatest difference in an impurity function with respect to the outcome measure. At each point in the process, the root (parent nodes) split into two child nodes. The Gini impurity function was used for this analysis, which uses the proportion of the dependent variable in the parent nodes and a weighted average of patients in the resulting child nodes to calculate impurity at each split [16,21,22]. The data continue to be split until at least one stopping rule is met. The final nodes, terminal nodes, are mutually exclusive and exhaustive.

For validation of trees, the dataset was divided into five subsets where each subset acts as a validation dataset while the remaining subsets are used as a training dataset. This process is repeated five times until each subset has acted as the validation dataset. All CART analyses were performed using SPSS Answer Tree 3.0 software [20].
